# IL‐17A contributes to HSV1 infection‐induced acute lung injury in a mouse model of pulmonary fibrosis

**DOI:** 10.1111/jcmm.13992

**Published:** 2018-10-30

**Authors:** Tao Chen, Hui Qiu, Meng‐Meng Zhao, Shan‐Shan Chen, Qin Wu, Nian‐Yu Zhou, Li‐Qin Lu, Jia‐Cui Song, Dan‐Li Tang, Dong Weng, Hui‐Ping Li

**Affiliations:** ^1^ Department of Respiratory Medicine School of Medicine Shanghai Pulmonary Hospital Tongji University Shanghai China; ^2^ Department of Respiratory Medicine School of Medicine Shanghai Pulmonary Hospital Soochow University Suzhou China

**Keywords:** acute exacerbations of idiopathic pulmonary fibrosis, endoplasmic reticulum stress, herpes simplex virus 1, interleukin‐17A

## Abstract

**Background:**

Patients with idiopathic pulmonary fibrosis (IPF) often experience acute exacerbation (AE) after an episode of common cold.

**Aims:**

To establish a mouse model of virus infection‐induced AE‐IPF and investigate the mechanism underlying the AE‐IPF.

**Methods:**

Herpes simplex virus 1 (HSV1) was inoculated intranasally to wild‐type (WT) and IL‐17A gene knockout (IL‐17A^‐/‐^) mice 21 days after intratracheal administration of bleomycin (BLM).

**Results:**

HSV1 infection caused acute exacerbation in mice with BLM‐induced fibrosis. Compared with the BLM+Saline mice, the mice with BLM+HSV1 showed significantly higher acute lung injury (ALI) score (*P *<* *0.0001), lower survival rate (100% vs 21.4%, *P < *0.0001), poorer lung function and higher inflammatory response representing by increased total inflammatory cells in bronchoalveolar lavage fluid (BALF) (*P *=* *0.0323), increased proportion of Th17 cells in peripheral blood (*P *=* *0.0004) and higher inflammatory factors in BALF. In addition, HSV1 infection increased the expression of endoplasmic reticulum stress (ERS)‐related proteins in mice with BLM‐induced fibrosis. The inhibition of ERS by tauroursodeoxycholic acid (TUDCA, an ERS inhibitor) significantly reduced the IL‐17A levels in BALF (*P *=* *0.0140) and TH17 cells in the peripheral blood (*P *=* *0.0084) of mice with BLM+HSV1, suggesting that suppression of ERS may reduce TH17 response in mice with AE‐IPF. Compared with WT mice with BLM+HSV1, IL‐17A^‐/‐^ mice with BLM+HSV1 had lower ALI score (*P *=* *0.0119), higher survival rate (78.6% vs 21.4%, *P *=* *0.004), improved lung function, and milder inflammatory response.

**Conclusions:**

HSV1 infection in addition to BLM‐induced IPF can successfully establish AE‐IPF in mice. IL‐17A and ERS promote lung inflammation in AE‐IPF development.

## 1 INTRODUCTION

Idiopathic pulmonary fibrosis (IPF) is chronic progressive fibrotic interstitial pneumonia with an unknown aetiology. Median survival time is between 2 and 3 years. The natural course of IPF shows several clinical characteristics: such as slow progression, long‐term stable stage and acute exacerbation of IPF (AE‐IPF). Approximately 1%‐20% of patients with IPF develop AE‐IPF within 1 year.^1^, ^2^, ^3^ The clinical presentation of AE‐IPF is characterized by acute respiratory difficulty within 30 days, lung histopathology showing diffuse alveolar damages in addition to fibrosis, >50% mortality rate and median survival time of only 2‐4 months.^1^, ^2^ AE‐IPF‐associated deaths account for 46% of the total deaths of patients with IPF.^1^ Thus, AE‐IPF is the leading cause of death in patients with IPF.

Whether infection can induce AE‐IPF remains elusive. Analyses using pan‐viral microarray and multiplex polymerase chain reaction have found the common respiratory viruses in the respiratory track of patients with AE‐IPF, such as cytomegalovirus, respiratory syncytial virus, parainfluenza virus, rhinovirus and coronavirus.^4^, ^5^ A previous epidemiological study has shown that AE‐IPF occurs frequently in winter and spring and in patients using immunosuppressants,^6^ indicating that virus infection may be a critical factor contributing to AE‐IPF. Studies using animal models have also demonstrated that mammal herpes virus γHV‐68 infection in addition to lung fibrosis can exacerbate the lung fibrosis.^7^, ^8^ However, animal models of infection‐induced AE‐IPF have not been established, which seriously limits the investigation on the biological mechanisms and therapeutic intervention of AE‐IPF.

In our previous studies, we used twice intratracheal administration of bleomycin (BLM) to successfully establish a mouse model of non‐infection‐induced AE‐IPF.^9^, ^10^ We found that IL17A levels in the BALF of mice with BLM+BLM‐induced AE‐IPF were increased significantly compared with mice with stable BLM‐induced IPF and intraperitoneal injection of anti‐IL‐17A antibody alleviated lung inflammation in the mice.^9^ In the current study, we aim to use human herpes simplex virus 1 (HSV1) to establish a mouse model of virus infection‐induced AE‐IPF, to investigate the role of IL‐17A in AE‐IPF by using IL‐17A gene knockout (IL‐17A^‐/‐^) mice and use the endoplasmic reticulum stress (ERS) inhibitor, tauroursodeoxycholic acid (TUDCA), to examine the contribution of ERS to AE‐IPF. The mouse model of virus infection‐induced AE‐IPF developed in this study can be used as an effective tool to develop and investigate new therapies for AE‐IPF.

## 2 MATERIALS AND METHODS

### 2.1 Animals

Specific pathogen‐free (SPF) grade wild‐type (WT) male C57BL/6 mice were purchased from Beijing Vital River Laboratory Animal Technology Co., Ltd. (Beijing, China), and male IL‐17A knockout (IL‐17A^‐/‐^) mice were obtained from Tokyo University of Science. The mice were housed in separated cages till 6‐8 weeks of age and allowed to access water and food freely. The protocols for mouse maintenance and experiments have been approved by the Institutional Animal Care and Use Committee of Tongji University (Approval No.: K17‐016). WT and IL‐17A^‐/‐^ mice were randomized into four groups: (a) acute exacerbation (BLM+HSV1); (b) stable fibrosis (BLM+Saline); (c) virus infection alone (Saline+HSV1); (d) normal control (Saline+Saline). For mechanistic investigation, WT mice were randomized into two additional groups: acute exacerbation (BLM+HSV1+Saline) and TUDCA treatment group (BLM+HSV1+TUDCA).

### 2.2 Mouse model of AE‐IPF

WT and IL‐17A^‐/‐^ mice were induced to develop AE‐IPF by two steps (Figure S1). (a) Bleomycin‐induced lung fibrosis: each mouse in the BLM+HSV1 and BLM+Saline groups was administered intratracheally with 4 mg/kg BLM (BLM from Dalian Meilun Biotech Co., Dalian, China) in 40 μL saline, and each mouse in the Saline+Saline and Saline+HSV1 groups was administered with the same volume of saline in the same manner. (b) Intranasal inoculation of HSV1to induce AE‐IPF: 21 days after the BLM administration and according to the previous description by McMillan et al,^8^ HSV1 virus stock (5 × 10^5^ PFU, provided by the Institute Pasteur of ShangHai Chinese Academy of Sciences, the viral titre used in the current study is based on our previous study^11^) was mixed with 40 μL DMEM media for the intranasal inoculation. Each mouse in the Saline+HSV1, BLM+HSV1, and BLM+HSV1+TUDCA groups was inoculated intranasally with 10 μL of the HSV1 suspension for four times, and each mouse in the Saline+Saline and BLM+Saline groups was administered with the equal volume of saline. According to the description by Keestra‐Gounder et al,^12^ each mouse in the BLM+HSV1+TUDCA group received daily intraperitoneal injection of TUDCA (250 mg/kg; Sigma‐Aldrich, Darmstadt, Germany) after the intranasal inoculation of HSV1. Bronchoalveolar lavage fluid (BALF) was collected on post‐HSV1‐infection day 3 (day 24 after the BLM administration) and day 7 (day 28 after the BLM administration) and centrifuged at 200 *g* for 5 minutes and the supernatant was harvested and stored at −80°C for future experiments. Left lung tissue was frozen at −80°C. Part of the right lung tissue was used to determine wet to dry weight ratio; the remaining right lung tissue was fixed in a standard tissue fixation solution (Wuhan Goodbio Technology Co. Ltd, Wuhan, China) for hematoxylin and eosin (H & E) and Masson staining. The mice used to collect the BALF and lung tissue were from two batches of experiments, but modelled in the same way. Therefore, the lung tissues used for wet to dry ratios and histopathology were not perfused by saline.

### 2.3 Lung tissue histopathological examination

After 24 hours of fixation, paraembedded lung tissue sections were prepared and used for H & E and Masson staining. The stained tissue sections were scanned by a Leica slide scanner (LEICA SCN400). According to the report by Mikawa et al,^13^ the acute lung injury (ALI) scores of the H&E staining tissue sections were determined. The Masson staining score was determined following the criteria for the estimation of lung fibrosis severity developed by Ashcroft et al.^14^ Two pathologists with extensive experience determined the ALI score and Masson staining score independently, and the average scores were used.

### 2.4 Survival curve

Twenty‐one days after the intratracheal administration of BLM in WT and IL‐17A^‐/‐^ mice, the mice were randomized into BLM+Saline and BLM+HSV1 groups (in total four groups: WT+BLM+Saline, WT+BLM+HSV1, IL‐17A^‐/‐^+BLM+Saline, and IL‐17A^‐/‐^+BLM+HSV1). Mouse survival was recorded starting from post‐HSV1 infection day 0 for 4 weeks (till day 28).

### 2.5 BALF protein content measurement

BALF supernatant was centrifuged at 800 *g* for 5 min to remove precipitate, and the supernatant was harvested for protein content measurement by the Bicinchoninic acid (BCA) assay. A microplate reader (BioTek Instruments, Inc., Winooski, VT) was used to read the BCA assay results at the wavelength of 560 nm.

### 2.6 Lung tissue wet/dry weight ratio

The wet weight of the middle lobe of the right lung was measured, and the dry weight was measured after the lung tissue was dried in a vacuum freeze dryer (LGJ‐10D; Beijing Sihuan Company, Beijing, China) overnight. Wet/dry weight ratio = wet weight ÷ dry weight.

### 2.7 Mouse lung function

Seven mice were randomly selected from each group at post‐HSV1 infection day 3 (Day 24) and day 7 (Day 28) and injected with 9 mL/kg 2% pentobarbital sodium (Bio‐Light Biotech, Zhuhai, China) intraperitoneally. After the mice were anaesthetized, a tracheal tube was placed to measure lung function using a mouse lung function analyser (Data Sciences International, Inc., ST. Paul, MN). Mouse vital capacity (VC), forced vital capacity (FVC), forced expiratory volume in the first 50 millisecond (FEV50) and dynamic pulmonary compliance (Cdyn) were recorded.

### 2.8 Flow cytometry

Cells were collected from BALF by centrifugation. The cells were counted by Coomassie brilliant blue staining and then suspended in the FACS solution. The FACS cell suspension was then divided into two halves. One half was used for labelling with antimouse CD11b‐FITC, F4/80‐APC and Gr‐1‐PE antibodies on cell surface. The other half was labelled with antimouse CD4‐FITC on cell surface first, and then the cells were fixed and permeabilized for cytoplasmic staining with antimouse IFN‐γ‐APC and IL‐17‐PE antibodies. All the fluorescence dye‐labelled antibodies were purchased from eBioscience (San Diego, CA). The antibody‐stained cells were analysed in a flow cytometer (Beckman Coulter, Brea, CA). Mouse peripheral blood was collected, and mononuclear cells (PBMCs) were isolated after erythrocytes were lysed. The PBMCs were analysed by flow cytometry in a similar manner.

### 2.9 Cytokine and chemokine measurement

Liquid suspension array technology was used to profile the cytokines and chemokines in BALF supernatants (n ≥ 5 mice from each group). The cytokine and chemokine list in the liquid suspension array includes IL‐1α, IL‐1β, IL‐2, IL‐3, IL‐4, IL‐5, IL‐6, IL‐9, IL‐10, IL‐12 (p40), IL‐12 (p70), IL‐13, IL‐17A, CC chemokine subfamily of eosinophil chemotactic proteins (Eotaxin), G‐CSF, GM‐CSF, INF‐γ, KC (CXCL1), monocyte chemoattractant protein 1 CCL2 (MCP‐1), macrophage inflammatory protein‐1β CCL4 (MIP‐1β), regulated on activation, normal T cell expressed CCL5 (RANTES) and TNF‐α (Bio‐Plex Mouse Cytokine 23‐Plex). Inflammatory factor IL‐23 was measured by enzyme‐linked immunosorbent assay (ELISA; Neobioscience, Shenzhen, China).

### 2.10 Western blot

Lung tissue was homogenized in a tissue homogenizer and then lysed thoroughly. The tissue lysate was centrifuged, and the supernatant was collected. Protein concentration of the supernatants was determined by BCA assay. For each sample, equal amount of proteins were loaded on a 10%‐15% SDS‐polyacrylamide gel. After electrophoresis, the proteins were transferred to a PVDF membrane. The membrane was probed with polyclonal antimouse antibodies against activating transcription factor 6 (ATF6), activating transcription factor 4 (ATF4), CCAAT/enhancer binding protein (C/EBP) homologous protein (CHOP) and β‐actin. All the antibodies were purchased from Abcam. After the membrane was probed with secondary antibodies, the chemiluminescent signals were developed and the signal intensity was measured.

### 2.11 Statistical analyses

The statistical analysis software Graphpad prism6 was used for data analyses. Continuous variables are presented as mean ± standard deviation. Two‐group comparison was analysed by independent sample *t* test. Multi‐group comparison was analysed by one‐way ANOVA and then Fisher's LSD test. The Kaplan‐Merier method was used to plot survival curve, and the survival time was compared by the log‐rank test. *P *< 0.05 was considered statistically significant.

## 3 RESULTS

### 3.1 HSV1 infection stimulated acute exacerbation of BLM‐induced IPF in mice

**Figure 1:**
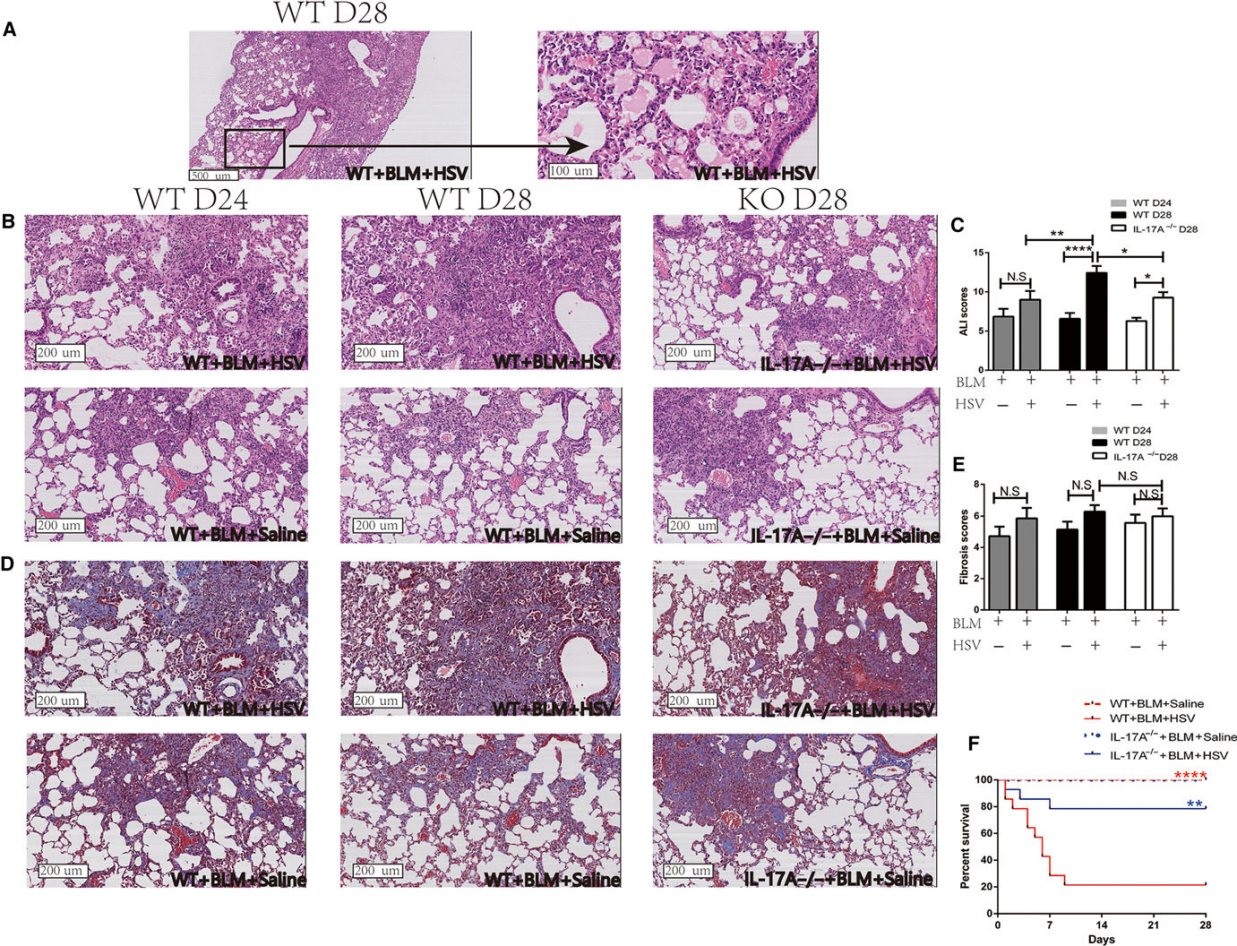
Mouse lung histopathology and survival rate. A, Representative images of H&E staining of the lung tissue on day 28 of mice in the BLM+HSV1 group (Left image: 40× magnification, scale bar = 500 μm. Right image: 200× magnification, scale bar = 100 μm). The 200× image shows lung interstitial congestion and oedema, damages in the alveolar structure and formation of transparent membrane. B, Representative images of H&E staining of the lung tissue of mice in each respective group (100× magnification, scale bar = 200 μm). On day 28, the inflammation surrounding the fibrotic tissues was the most severe in the BLM+HSV1 group. Inflammatory cell infiltration in the lung tissue reduces in IL‐17A‐/‐ mice. C, Mouse lung tissue ALI score. D, Representative images of Masson staining (100× magnification, scale bar = 200 μm). Collagen deposition is in the peribronchial area and alveolar septum in both WT and IL‐17A‐/‐ mice. BLM or saline were administered intratracheally to mice, and 21 days after the BLM administration, saline or HSV1 solution was inoculated intranasally to the mice. On postHSV1‐infection day 3 and day 7, mouse lung tissue was collected for H&E and Masson staining (n = 5 in each group). E, Mouse lung tissue fibrosis score. F, survival rate of WT and IL‐17A‐/‐ mice in the BLM+HSV1 and BLM+Saline groups. n = 14 in each group for survival observation. *P < 0.05, **P < 0.01, ***P < 0.001, ****P < 0.0001. NS, not significant

H&E staining revealed damages in alveolar structure and widening of alveolar septum in the mice treated with BLM (Figure 1A,B). Lung tissue inflammation in the WT+BLM+HSV1 group was worse than that in the WT+BLM+Saline and the worst on day 28. On day 28, the WT+BLM+HSV1 mice presented typical AE‐IPF characteristics, including alveolar septal congestion and oedema, inflammatory cell infiltration in the lung tissue, alveolar epithelial damages and apparent transparent membrane structure in the alveolar cavity (Figure 1A). The IL‐17A^‐/‐^+BLM+HSV1 mice showed less inflammatory cell infiltration in the lung tissue than the WT+BLM+HSV1 mice (Figure 1B). The BLM+HSV1 group had a significantly higher ALI score than the BLM+Saline group (*P *< 0.0001, Figures 1C, S5), and the IL‐17A^‐/‐^+BLM+HSV1 mice showed significantly lower ALI score than the WT+BLM+HSV1 mice (*P *=* *0.0159, Figure 1C).

BLM+HSV1 mice developed severe inflammation and formed dense aggregates of inflammatory cells after 1 week of HSV1 infection, which looks like the fibrosis foci, thus, Masson staining seemed as if the WT+BLM+HSV1 mice might develop more severe fibrosis than the WT+BLM+Saline mice (Figure 1D), but the fibrosis score was not statistically significantly different (Figure 1E). In addition, hydroxyproline content measurement also demonstrated similar results (Figure S2). These data suggest that HSV1 infection may stimulate acute lung damages in short‐term but may not exacerbate BLM‐induced fibrosis substantially. In addition, HSV1 infection alone can also cause acute inflammation in lung tissue, which is IL‐17‐dependent (Figure S4).

Survival of WT and IL‐17A^‐/‐^ mice was observed from day 0 (the day when HSV1 was inoculated intranasally) for 4 weeks. The survival rate of WT+BLM+HSV1 mice (21.4%, 3/14) was significantly lower than that of WT+BLM+Saline group (100%, 14/14, *P *< 0.0001, Figure 1F). Notably, the survival rate of IL‐17A^‐/‐^+BLM+HSV1 mice (11/14, 78.6%) was significantly higher than that of WT+BLM+HSV1 mice (*P *=* *0.004, Figure 1F).

**Figure 2:**
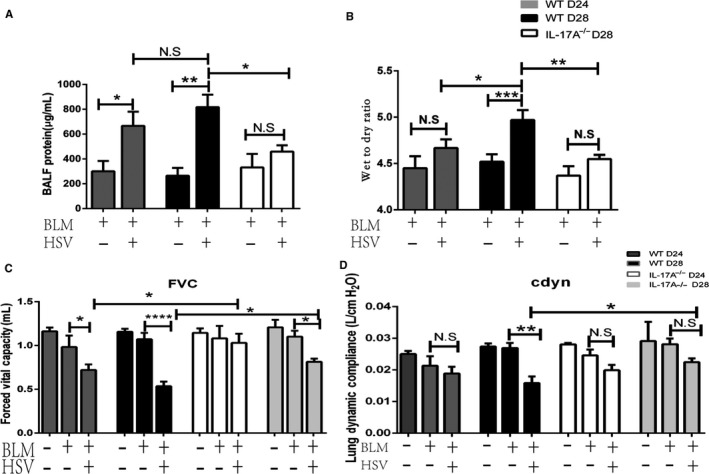
HSV1 infection in addition to BLM‐induced lung fibrosis impaired alveolar structure and lung function. A, Total protein in BALF. On day 24 (*P* = 0.0189) and day 28 (*P* = 0.0019), the total protein in BALF is significantly higher in the BLM+HSV1 group than in the BLM+Saline group. B, Lung tissue wet/dry weight ratio. On day 28, lung oedema is the most severe in the BLM+HSV1 group. The ratio is smaller in the IL‐17A‐/‐+BLM+HSV1 group than in the WT+BLM+HSV1 group (*P* = 0.0051). C, FVC. On day 24 (*P* = 0.0177) and day 28 (*P *< 0.0001), FVC is lower in the BLM+HSV1 group than in the BLM+Saline group. FVC is higher in the IL‐17A‐/‐+BLM+HSV1 group than in the WT+BLM+HSV1 group (*P* = 0.0054). D, Cdyn. On day 28, Cdyn is lower in the BLM+HSV1 group than in the BLM+Saline group (*P* = 0.0012) and is better in the IL‐17A‐/‐+ BLM+HSV1 group than in the WT+BLM+HSV1 group (*P* = 0.0254). For the statistical analysis of lung function, n = 7 in each group. For other statistical analyses, n = 5 in each group. *P < 0.05. **P < 0.01. ***P < 0.001. ****P < 0.0001. NS, not significant; FVC, force vital capacity; Cdyn, dynamic pulmonary compliance

The total protein in BALF in the BLM+HSV1group was significantly increased than that in the BLM+Saline group (Figure 2A), and so was the lung tissue wet/dry weight ratio (Figure 2B). Compared with WT+BLM+HSV1 mice, IL‐17A^‐/‐^+BLM+HSV1 mice had significantly reduced total protein in the BALF (*P *=* *0.0358, Figure 2A) and lung tissue wet/dry weight ratio (Figure 2B). These data indicate that HSV1 infection can cause lung oedema, alveolar epithelial damages and protein leakage from alveoli and that IL‐17A^‐/‐^ appear to attenuate the HSV1‐stimulated adverse effects.

On post‐HSV1 infection day 3, the FVC of the BLM+HSV1 group decreased significantly compared with that in the BLM+Saline group (Figure 2C), but Cdyn remained similar in the two groups (*P *=* *0.4259, Figure 2D). On post‐HSV1 infection day 7, both FVC and Cdyn of the BLM+HSV1 group reduced significantly compared with those of the BLM+Saline group. The extent of lung functional deterioration was significantly milder in the IL‐17A^‐/‐^+BLM+HSV1 mice than in the WT+BLM+HSV1 mice.

### 3.2 TH17 response played critical roles in AE‐IPF

**Figure 3:**
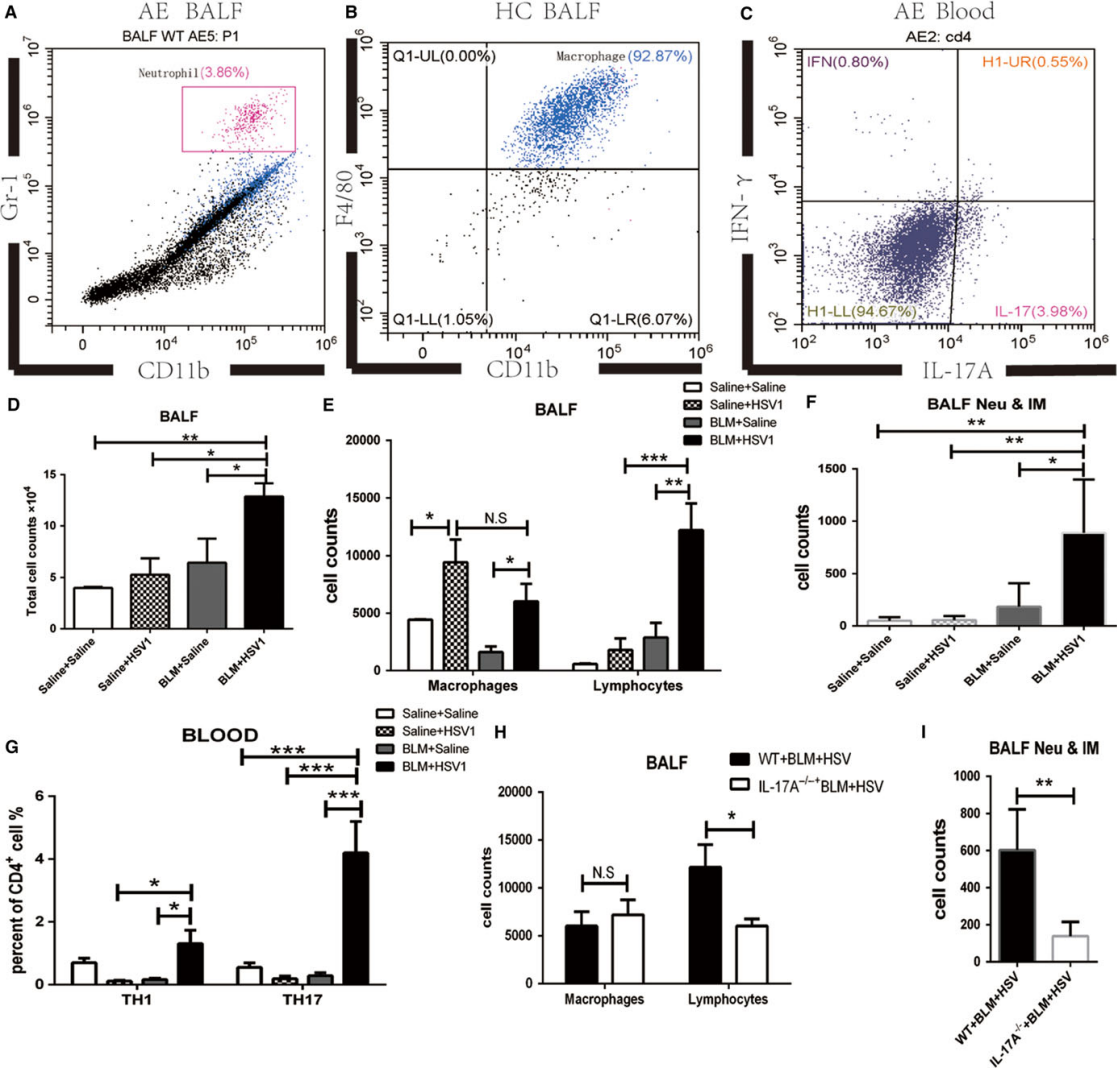
TH17 was associated with BLM+HSV1‐induced acute inflammation in lung tissue. Granulocyte‐macrophages and lymphocytes were gated by their feature of FSC‐A and SSC‐A. A, Neutrophils were defined as CD11b+ and Gr‐1+. B, Macrophages were defined as CD11b+ and F4/80+. Macrophages were the main immunocytes in BALF of Saline+Saline mice. C, CD4+ lymphocytes were isolated by FITC‐labelled antibodies. Anti‐INF‐γ and IL‐17A antibodies were used to label TH1 and TH17 cells, respectively. D, Total number of cells in BALF. Total number of cells is greater in the BLM+HSV1 group than in BLM+Saline group (*P* = 0.0323). E and F, The number of different types of inflammatory cells in BALF. The numbers of macrophages (*P* = 0.0288), lymphocytes (*P *= 0.0016) and Neu & IM (*P* = 0.0108) are significantly greater in the BLM+HSV1 group than in the BLM+Saline group. G, The proportions of TH1 and TH17 cells in the CD4+ lymphocytes of peripheral bloods. The proportion of Th17 in CD4+ T cells is higher in the BLM+HSV1 group than in the BLM+Saline group (*P* = 0.0004). H and I, The numbers of different types of inflammatory cells in the BALF of WT and IL‐17A‐/‐ mice in the BLM+HSV1 group on day 28. The numbers of lymphocytes (*P* = 0.0463) and Neu & IM (*P* = 0.0294) in the BALF of IL‐17A‐/‐ mice are lower than those of WT mice. n = 5 in each group. Mice were infected with HSV1 by intranasal inoculation 21 days after the intratracheal administration of BLM. On post‐HSV1 infection day 7, mononuclear cells were collected from peripheral bloods and BALF. Neu & IM: neutrophils and inflammatory monocytes. *P < 0.05. **P < 0.01. ***P < 0.001. ****P < 0.0001. NS, not significant

On post‐HSV1 infection day 7 (Day 28), inflammatory cells were collected from peripheral blood and BALF, and the proportions of inflammatory monocytes & neutrophils (Figures 3A, S3 Neu & IM: neutrophils and inflammatory monocytes), macrophages (Figures 3B, S3), TH1 cells (Figures 3C, S3) and TH17 cells (Figures 3C, S3) were analysed by flow cytometry. The total number of inflammatory cells in BALF was significantly higher in the BLM+HSV1 group than in the BLM+Saline group (Figure 3D). After mice were inoculated with HSV1 (the Saline+HSV1 and BLM+HSV1group), the number of macrophages in BALF of the Saline+HSV1 and BLM+HSV1groups increased significantly (Figure 3E) to fight the virus infection. Compared with the macrophage increase in the healthy mice after the HSV1 infection, HSV1‐stimulated inflammatory monocytes and neutrophils (Figure 3F) and lymphocyte (Figure 3E) recruitment were more apparent in the BLM‐treated mice, indicating that inflammatory monocytes and neutrophils and lymphocyte recruitment in the lung may be associated with acute lung damage.

The proportion of TH17 cells in the CD4+ T cells of the peripheral blood of BLM+HSV1 group increased significantly compared with that of the other three groups (Figure 3G), and the proportion of TH1 cells also significantly increased in the BLM+HSV1 than in the BLM+Saline groups (*P *=* *0.0194, Figure 3G). On day 28, the IL‐17A^‐/‐^+BLM+HSV1 mice had significantly fewer lymphocytes (Figure 3H) and inflammatory monocytes & neutrophils (Figure 3I) in the BALF than the WT+BLM+HSV1 mice. These results indicate that BLM+HSV1‐induced acute lung damages could be associated with TH17‐promoted inflammatory monocyte and neutrophil chemotaxis towards the lung.

**Figure 4:**
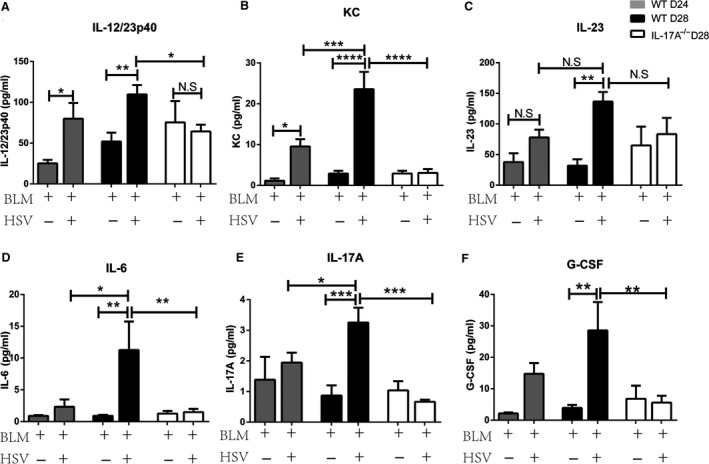
HSV1 infection in addition to BLM‐induced lung fibrosis resulted in IL‐17A‐associated acute inflammation in the lung. A, IL‐12/23p40. B, KC. C, IL‐23. D, IL‐6. E, IL‐17A. F, G‐CSF. Inflammatory factor concentration in BALF supernatants was analysed by liquid suspension array technology. IL‐23 concentration was measured by ELISA. On day 24, compared with the BLM+Saline group, the BLM+HSV1 group shows significantly higher 12/23p40 (*P* = 0.0327, A) and KC (*P* = 0.0327, B). On day 28, compared with the BLM+Saline group, the BLM+HSV1 group shows significantly higher 12/23p40 (*P* = 0.0005, A), KC (*P* < 0.0001, B), IL‐23 (*P* = 0.0014, C), IL‐6 (*P* = 0.0017, D), IL‐17A (*P* = 0.0067, E) and G‐CSF (*P* = 0.0106, F). IL‐17A‐/‐+BLM+HSV1 mice show significantly lower IL‐12/23p40 (*P* = 0.0258, A), KC (*P* < 0.0001, B), IL‐6 (*P* = 0.0188, D), IL‐17A (*P* = 0.0001, E) and G‐CSF (*P* = 0.0045, F) than WT+BLM+HSV1 mice. n = 5 in each group. *P < 0.05. **P < 0.01. ***P < 0.001. ****P < 0.0001. NS, not significant

Compared with the BLM+Saline group, the BLM+HSV1 group demonstrated significantly increased IL‐12/23 P40 (Figure 4A) and KC (Figure 4B) on day 24 and elevated IL‐12/23p40, KC, IL‐23 (Figure 4C), IL‐6 (Figure 4D), IL‐17A (Figure 4E) and G‐CSF (Figure 4F) on day 28. These data suggest that HSV1 infection may cause acute pulmonary inflammation and consequently increase inflammatory factor concentration in the BALF. The inflammatory factor increase was the highest on day 28. These results are consistent with the histopathology results. On day 28, the IL‐17A^‐/‐^+BLM+HSV1 mice had significantly lower IL‐12/23p40, KC, IL‐6 and G‐CSF compared with the WT+BLM+HSV1 mice, indicating that IL‐17A may contribute to the HSV1‐induced acute inflammation in fibrotic lung.

### 3.3 HSV1 infection exacerbated endoplasmic reticulum stress (ERS) in fibrotic lung tissue

**Figure 5:**
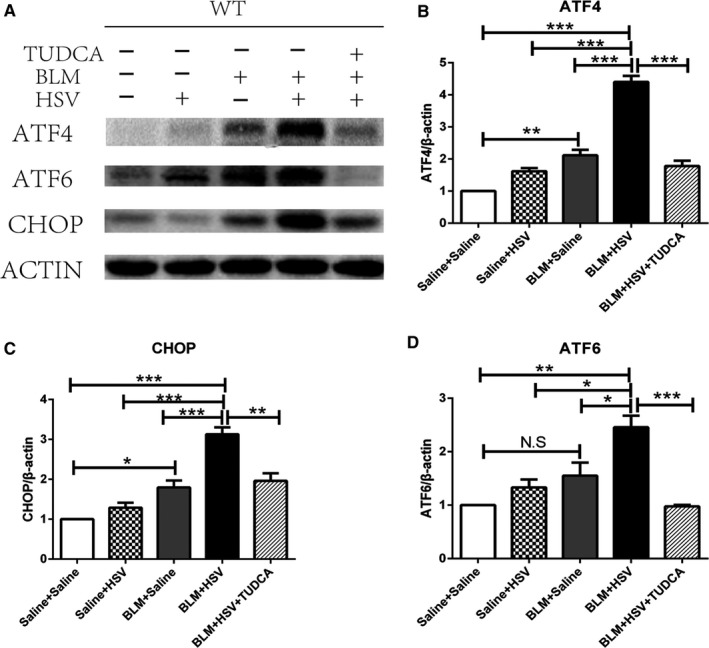
ERS was activated in the lung tissue of mice with AE‐IPF. A, Representative images of immunoblot. B‐D, Protein densitometry analysis of ATF4 (B), CHOP (C) and ATF6 (D). Expressions of the three proteins in the BLM+HSV1 group are significantly higher than those in the other four groups. Saline or HSV1 solution was administered intranasally to the mice 21 days after the intratracheal administration of BLM, one group of BLM+HSV1 mice was administrated with TUDCA. On post‐ HSV1 infection day 7, protein was extracted from the lung tissues. Antimouse ATF4, CHOP, ATF6 and actin antibodies were used for immunoblotting assay. The protein signals were visualized by chemiluminescence. n = 4 in each group. *P < 0.05. **P < 0.01. ***P < 0.001. ****P < 0.0001. NS, not significant

Endoplasmic reticulum stress (ERS) occurs commonly in the alveolar epithelial cells of patients with IPF.^15^, ^16^ ERS‐associated proteins were all overexpressed in the fibrotic lung tissues (Figure 5A). The BLM+Saline mice expressed significantly higher levels of ATF4 (*P *=* *0.0018, Figure 5B) and CHOP (*P *=* *0.0223, Figure 5C) in the lung tissues than the Saline+Saline mice. The BLM+HSV1 group demonstrated significantly higher expression of ATF4 (*P *< 0.0001), CHOP (*P *=* *0.0005) and ATF6 (*P *=* *0.0257, Figure 5D) than the BLM+Saline mice. TUDCA is an inhibitor of ERS. When we used TUDCA to treat BLM+HSV1 mice, the BLM+HSV1+TUDCA group expressed significantly lower levels of ATF‐4 (*P *< 0.0001), CHOP (*P = *0.0011) and ATF6 (*P *< 0.0001) than the BLM+HSV1 mice. These data indicate that ERS may be activated in BLM‐induced lung fibrosis and be further stimulated by HSV1 infection‐induced AE‐IPF and TUDCA can attenuated the ERS in AE‐IPF model.

### 3.4 The ERS inhibitor TUDCA reduced IL‐17A production and attenuated the lung tissue damage during AE‐IPF

**Figure 6:**
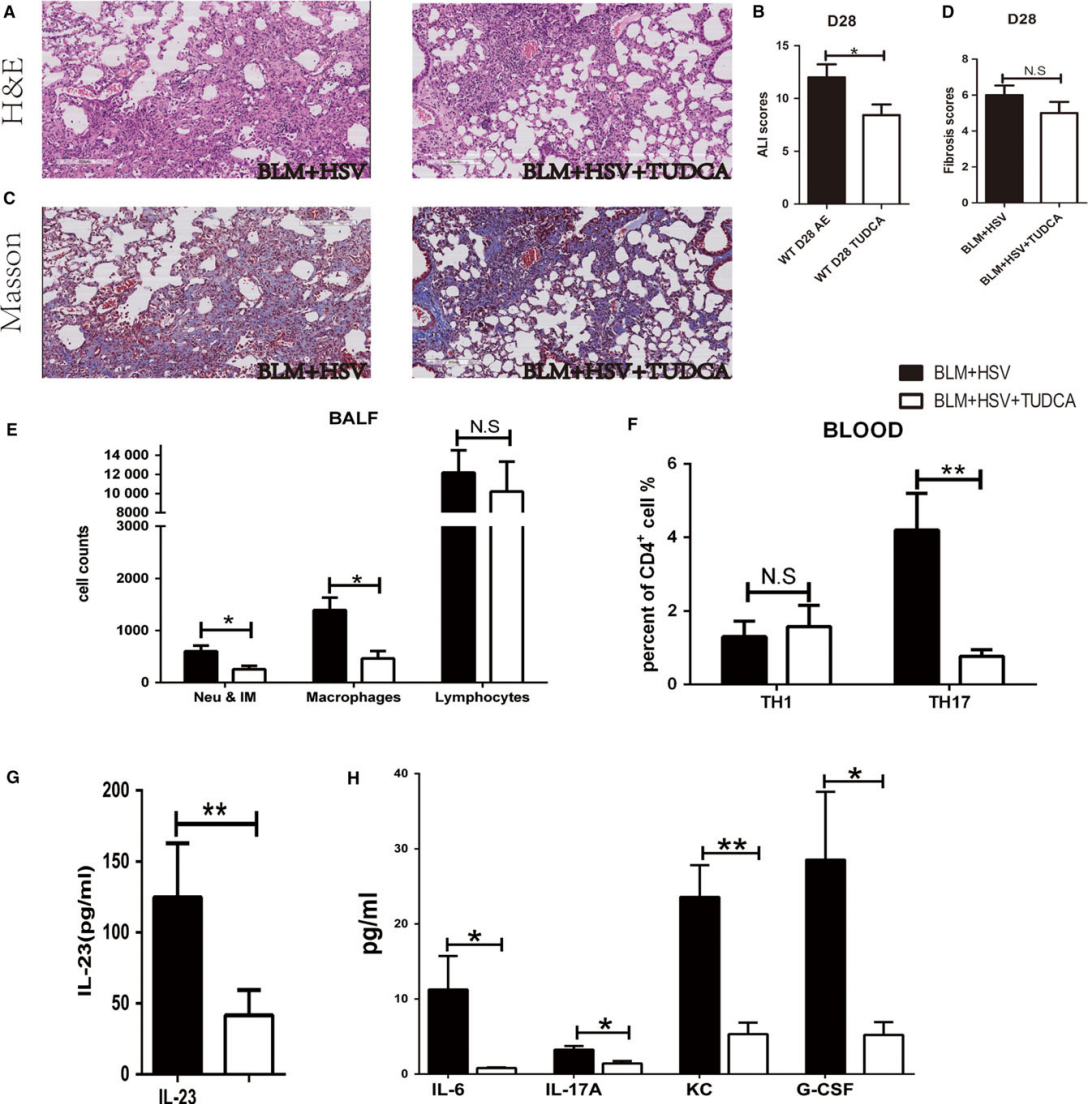
The ERS inhibitor TUDCA significantly attenuated BLM+HSV1‐induced acute inflammation in the lung. A, Representative images of H&E staining of the BLM+HSV1+Saline and BLM+HSV1+TUDCA groups (100× magnification, scale bar = 200 μm). TUDCA treatment reduces inflammatory cell infiltration in lung tissue but does not affect the extent of lung fibrosis. B, ALI score. C, Masson staining. D, Fibrosis score. TUDCA treatment group shows lower ALI score than the control group (*P* = 0.0439) but similar fibrosis score. E, The numbers of different types of inflammatory cells in BALF. TUDCA treatment group has lower numbers of Neu & IM (*P* = 0.0451) than the control group. F, The proportion of TH1/TH17 in CD4+ T cells of peripheral blood. TUDCA treatment group shows significantly less TH17 cells (*P* = 0.0084). G and H. Inflammatory factor concentration in the BALF. TUDCA treatment reduces the concentrations of IL‐23 (*P* = 0.0053), IL‐6 (*P* = 0.0473), IL‐17A (*P* = 0.0140), KC (*P* = 0.0041) and G‐CSF (*P* = 0.0388). Saline or HSV1 solution was administered intranasally to the mice 21 days after they received the intratracheal administration of BLM. After the HSV1 infection, TUDCA (250 mg/kg) or saline was injected intraperitoneally daily for 7 days, and then lung tissue, blood and BALF were collected 7 days after the HSV1 infection. n = 5 in each group. Neu & IM: neutrophils and inflammatory monocytes. *P < 0.05. **P < 0.01. ***P < 0.001. ****P < 0.0001. NS, not significant

H&E staining revealed that the BLM+HSV1+TUDCA group showed less inflammatory cell infiltration in the alveolar septum (Figure 6A), milder lung tissue inflammation, lower ALI score (Figure 6B) than the BLM+HSV1+Saline group, whereas the severity of lung fibrosis remained similar in the two groups (Figure 6C,D). The BALF of the BLM+HSV1+TUDCA group contained significantly fewer inflammatory monocytes and neutrophils (Figure 6E), lower proportion of Th17 cells in the peripheral blood (Figure 6F) and reduced levels of IL‐23 (Figure 6G,), IL‐6, IL‐17A, G‐CSF and KC (Figure 6H) compared with the BALF of the BLM+HSV1+Saline group. These results support that TUDCA can attenuate BLM+HSV1‐induced acute lung injury and the molecular mechanism underlying the beneficial effects may be associated with the inhibition of TH17 response and IL‐17A production.

## 4 DISCUSSION

The recent published diagnostic criteria for AE‐IPF no longer emphasize the exclusion of infection,^1^ and evidence of virus infection has been found from the lung tissues of patients who died from AE‐IPF.^17^ To investigate the role of virus infection in AE‐IPF, we established a mouse model of HSV1‐induced AE‐IPF. HSV1 was inoculated intranasally to the mice that already developed BLM‐induced lung fibrosis. One week after the HSV1 infection, the mice showed obvious diffuse lung injury, higher ALI score than mice with stable BLM‐induced lung fibrosis, exacerbated lung oedema, increased protein contents in the alveolar cavity, diminished lung function and reduced survival. These HSV1 infection‐induced histological and functional exacerbations in mice with BLM‐IPF resemble the clinical presentations of patients with AE‐IPF, such as diffuse lung injury, diminished lung function and reduced survival rate, supporting that BLM+HSV1 treatment can mimic AE‐IPF development closely. Previous studies have shown that nasal inoculation of γHV68 virus in addition to lung fibrosis results in lung infection‐stimulated exacerbation of the lung fibrosis.^7^, ^8^ Notably, because γHV68 is a mammalian virus that infects animals but not humans, γHV68 infection in addition to lung fibrosis may not mimic the AE‐IPF development in human. A previous study administered lipopolysaccharides (LPS) intratracheally to mice 7 days after BLM‐induced lung fibrosis to mimic postoperative acute exacerbation in patients with interstitial lung disease.^18^ However, operation‐induced AE‐IPF occurs rarely in patients. In the current study, to establish AE‐IPF mouse model, we used HSV1 virus, which can infect humans easily, to infect mice that already developed stable BLM‐IPF. Our mouse model of AE‐IPF can better mimic the AE‐IPF development in patients.

Acute pulmonary inflammation is closely correlated with IL‐17A.^19^ IL‐17A, a pro‐inflammatory factor, is secreted predominantly by TH17 cells. IL‐17A in lung tissues enhances inflammatory responses by promoting inflammatory monocyte and neutrophil chemotaxis so to clear pathogens effectively.^20^ However, excessive inflammatory responses may damage tissue.^21^ Both neutrophils and inflammatory monocytes are involved in pathogen elimination and can cause acute lung inflammation in an IL‐17‐dependent manner.^20^ In the current study, the data indicates that IL‐17A may not play an important role in driving lung fibrosis at day 28 in mice in response to bleomycin treatment (Figure 1E). This data seems to be in contradiction to the work by Wilson et al^22^ that shows IL‐17A knockout mice are refractory to bleomycin‐induced lung fibrosis. This might be explained by the demonstrated fact that the formation of obvious lung fibrosis appears between 14 and 28 days after a single‐dose administration of bleomycin in mice,^23^ however, Wilson and colleagues drew the conclusion by evaluating fibrosis in 7 days after administrating BLM and this has been proved to be an acute inflammatory period. In contrast, we observed the similar degree of fibrosis and hydroxyproline content in WT+BLM+Saline and IL‐17A^‐/‐^+BLM+Saline groups in 28 days after administration of BLM. These suggested that IL‐17A might contribute to acute lung injury but not fibrosis in our study. Sandra et al^24^ showed similar conclusion in their previous study. They found that in experimental silicosis the acute alveolitis induced by silica is IL‐17A dependent, but this cytokine appears dispensable for the development of the late fibrotic lung responses to silica. Compared with the mice treated with BLM+Saline, the BALF of mice treated with BLM+HSV1 contained higher levels of IL‐6 and IL‐23, which may promote pathogenic IL‐17A^+^ T cell maturation^25^ and may ultimately increase the proportion of Th17 cells in peripheral blood. As far as we know, some innate immune cells such as γδT and ILC3 cells can also produce IL‐17A in the innate primary immune responses.^24^ In our preliminary study, we found that patients with AE‐IPF had higher TH17 levels in peripheral blood than patients with stable‐IPF (data not shown). Thus, in the current study, we focused on the roles of Th17 cells in the AE‐IPF model. In our current study, we cannot exclude the effects of IL‐17A produced by other cells such as γδT and ILC3 cells, because compared to WT mice, IL1‐7A^‐/‐^ mice might have different levels of IL‐17A‐producing γδT cells and ILC3 cells.

TH17 cells can release a large amount of IL‐17A to promote G‐CSF and KC secretion, and G‐CSF can mobilize neutrophils from the bone marrow to peripheral blood.^26^ KC can induce the chemotaxis of pro‐inflammatory cells in particular neutrophils to the lung,^27^ consequently increasing inflammatory monocytes and neutrophils and lymphocytes in the BALF of the mice with AE‐IPF and exacerbating the local inflammatory responses in the lung. Compared with the WT mice, the IL‐17A^‐/‐^ mice showed reduced G‐CSF and KC concentrations in the BALF, fewer inflammatory monocytes and neutrophils in the BALF, attenuated lung inflammation and dysfunction and higher survival rate. Although IL‐17A^‐/‐^+BLM+HSV1 mice showed significantly higher ALI score than the IL‐17A^‐/‐^+BLM+Saline mice, the total protein in BALF of the two groups did not show significant differences. IL‐17A knockout leads to a reduction in the number of infiltrated inflammatory monocytes and neutrophils in BALF. Fewer inflammatory monocytes & neutrophils in the BALF of IL‐17A^‐/‐^+BLM+HSV1 group could result in milder HSV1 infection‐induced alveolar epithelial injury and thus less plasma protein exudation. On the other hand, total protein in BALF is just one of the indexes to evaluate ALI. As reported by Mikawa et al,^13^ ALI score mainly emphasizes the levels of inflammatory cell infiltration. IL‐17A knockout obviously reduced the number of inflammatory monocytes and neutrophils in BALF (Figure 3I). However, inflammatory monocytes and neutrophils only accounted for a small proportion of inflammatory cells (0.167%) in the BALF of IL‐17A^‐/‐^+BLM+HSV1 mice. Other inflammatory cells might contribute to a higher ALI score. Nevertheless, IL‐17A^‐/‐^+BLM+HSV1 mice showed significantly lower ALI score than the WT+BLM+HSV1 mice (Figure 1C). Thus, IL‐17A knockout appears to partially relieve the HSV1 infection‐induced acute lung injury in BLM+HSV1 mice. Our results showed that IL‐17A knockout might not influence the clearance of HSV virus (Figure S6A,B). Bagri and colleges also reported that HSV viral titres in IL‐17A^‐/‐^ mice were not significantly different from that of WT mice during genital HSV infection.^28^ However, neither our results nor Bagri's work were actual measurements from the lung. More work should be done to investigate the effects of IL‐17A on the clearance of HSV by detecting the actual viral titres from lung by standard methods such as plaque assay or qRT‐PCR in the future. Above all, we hypothesize that IL‐17A could be the key mediator in the HSV1 infection‐induced excessive acute inflammation in fibrotic lung.

**Figure 7:**
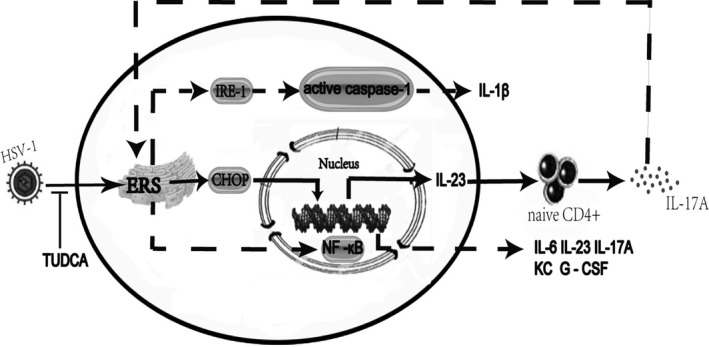
Cartoon diagram showing the hypothesis that IL‐17A and ERS contribute to HSV1 infection‐induced AE‐IPF. We concluded three possible mechanisms in this model. In mouse lung tissue, BLM‐mediated lung fibrosis stimulates ERS. (1) In addition to the existing ERS, HSV1 infection further exacerbates ERS and increases CHOP expression. CHOP, as a transcription factor, elevates the secretion of inflammatory factors, such as IL‐ 23. IL‐23 then promotes IL‐17A release. IL‐17A can enhance ERS by a positive feedback mechanism so to induce acute inflammatory reactions and acute lung damages. (2) Exacerbated ERS might increase IRE‐1 expression, resulting in caspase‐1 cleavage and mature IL‐1β production. This enhanced IL‐1β secretion stimulates further IL‐17A production from CD4+ effector T cells through IL‐1 receptor signalling. (3) ER stress increases cytokine secretion (IL‐6, IL‐17A, IL‐23, KC and G‐CSF) by activating NF‐κB. These cytokines contribute to HSV1‐induced acute inflammation in fibrotic lung

In ERS response, protein folding is disrupted, which leads to the accumulation of unfolded protein in the endoplasmic reticulum (ER) and the consequent development of unfolded protein response (UPR) in the ER.^29^ Moderate or normal UPR can reduce the accumulation of unfolded protein in the ER and restore the normal physiological function of ER. However, excessive UPR may trigger inflammatory responses.^30^ A previous study has found the concurrence of herpes virus infection and UPR in the alveolar epithelial cells of patients with IPF, suggesting that virus infection may contribute to alveolar epithelial injury by exacerbating ERS.^15^ The current study found that compared with the mice treated with BLM+Saline, the mice treated with BLM+HSV1 demonstrated significantly elevated expression of UPR‐related proteins and particularly higher CHOP expression in lung tissue. The three signalling pathways involved in UPR: PERK, IRE‐1, and ATF6, can all induce CHOP overexpression.^31^ In our study, ATF4 and ATF6 overexpression was quite obvious in the mice treated with BLM+HSV1. Jane and colleagues have found that CHOP can act as a transcription factor to co‐ordinate NF‐κB to stimulate IL‐23p19 expression synergistically.^32^ Our previous study has demonstrated that ERS occurs in AEC. In our previous study, we used alveolar epithelial cells (A549) to establish AE‐IPF cell model, and found that HSV1 infection stimulated the ER stress pathways in AEC in a time‐ and dose‐dependent manner.^11^ In the current study, the mice treated with BLM+HSV1 had increased IL‐23 levels in the BALF compared with the mice treated with BLM+Saline. IL‐23 binds to the IL‐23 receptor on naïve CD4^+^T cells to induce the CD4^+^ T cells to differentiate into TH17 cells, consequently increasing IL‐17A concentration.^33^, ^34^ In addition, we found when ERS was inhibited by TUDCA, the proportion of Th17 cells in mouse peripheral blood was reduced; IL17A concentration in BALF was decreased; acute lung injury was also attenuated. Our findings indicate that inhibition of ERS can alleviate lung inflammation, and it probably takes effects by reducing IL‐17A production. However, a previous study on respiratory syncytial virus infection in animal models suggests that ERS in lung tissue increases IRE‐1expression, resulting in caspase‐1 cleavage and mature IL‐1β production. This enhanced IL‐1β secretion might account for the aggravation of inflammation.^35^ While in a report on LPS‐induced acute lung injury, ERS can activate NF‐κB,^36^ then it can promote kinds of cytokines’ secretion, such as IL‐6, IL‐23, IL‐17A, KC and G‐CSF. Till now there has not been a study identifying which pathway plays a critical role, more work should be done to explore the relationship between ER stress and inflammation. In addition, the study also suggests that anti‐IL‐17A antibody treatment and IL‐17A gene knockout alleviate UPR in lung tissue substantially, supporting that IL‐17A may promote ERS in lung tissue^36^ (Figure 7).

## 5 CONCLUSIONS

This study successfully established HSV1 infection‐induced AE‐IPF in mice with BLM‐induced lung fibrosis and found that IL‐17A and ERS played key roles in the acute lung injury in AE‐IPF. Our study provides an animal model of AE‐IPF that highly mimic human AE‐IPF to facilitate the investigation of the aetiology, pathogenic mechanism and intervention of AE‐IPF.

## CONFLICT OF INTEREST

The authors confirm that there are no conflicts of interest.

## AUTHOR CONTRIBUTIONS

HP Li, D Weng, T Chen, H Qiu and MM Zhao participated in the conception, hypothesis and design of the study. T Chen and H Qiu performed the experiments. T Chen, H Qiu and MM Zhao carried out the statistical analyses. All authors contributed to interpretation of the data. T Chen and HP Li wrote the manuscript and all authors made critical revisions. All authors read and approved the final manuscript.

## ETHICAL STATEMENT

The study was approved by institutional ethics committee of Shanghai Pulmonary Hospital (No. K17‐016).
