# Supplementary material for: IL‐17A contributes to HSV1 infection‐induced acute lung injury in a mouse model of pulmonary fibrosis
Source: J Cell Mol Med. 2018 Oct 30;23(2):908–19. doi: 10.1111/jcmm.13992 (PMC6349191; doi:10.1111/jcmm.13992)
Supplement: Supplementary file 1 [file JCMM-23-908-s001.docx]

**
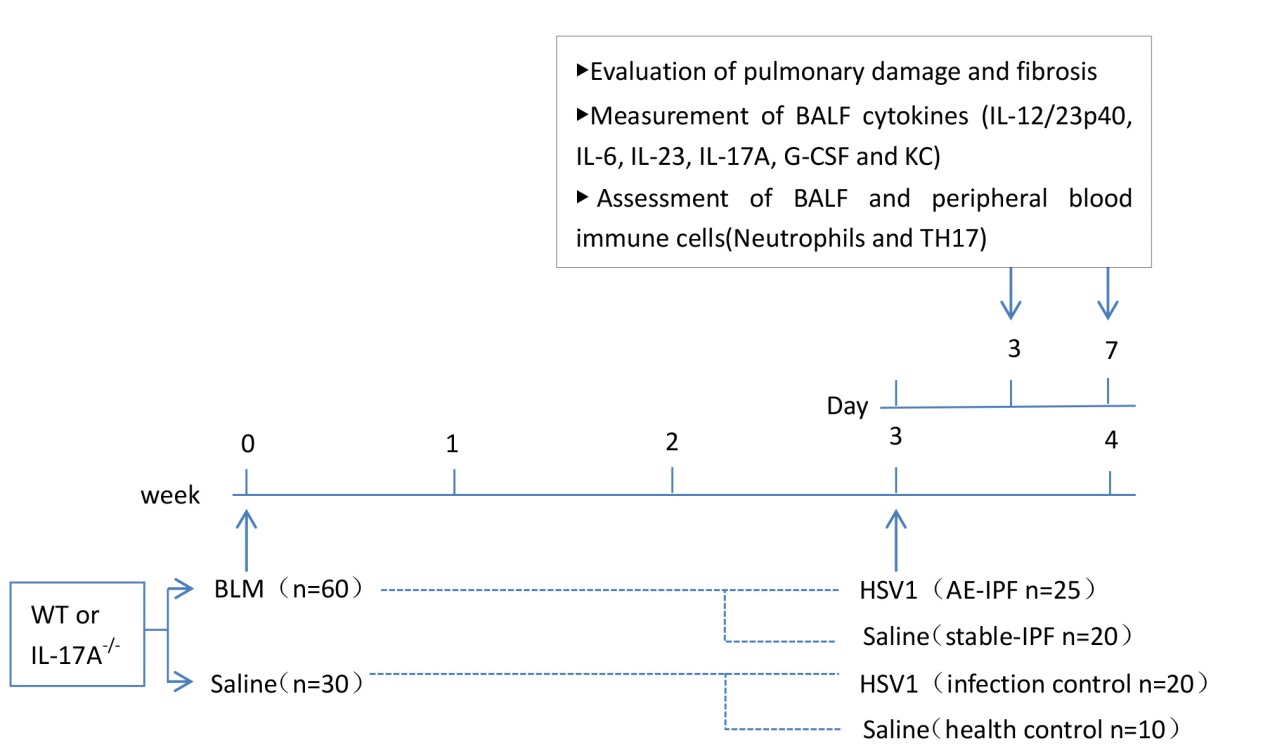
**

**Figure S1 The diagram illustrating the establishment of the mouse model of HSV1 infection-induced AE-IPF.**

BALF: Bronchoalveolar lavage fluid; BLM, bleomycin; HSV1: **herpes simplex virus 1.**

**
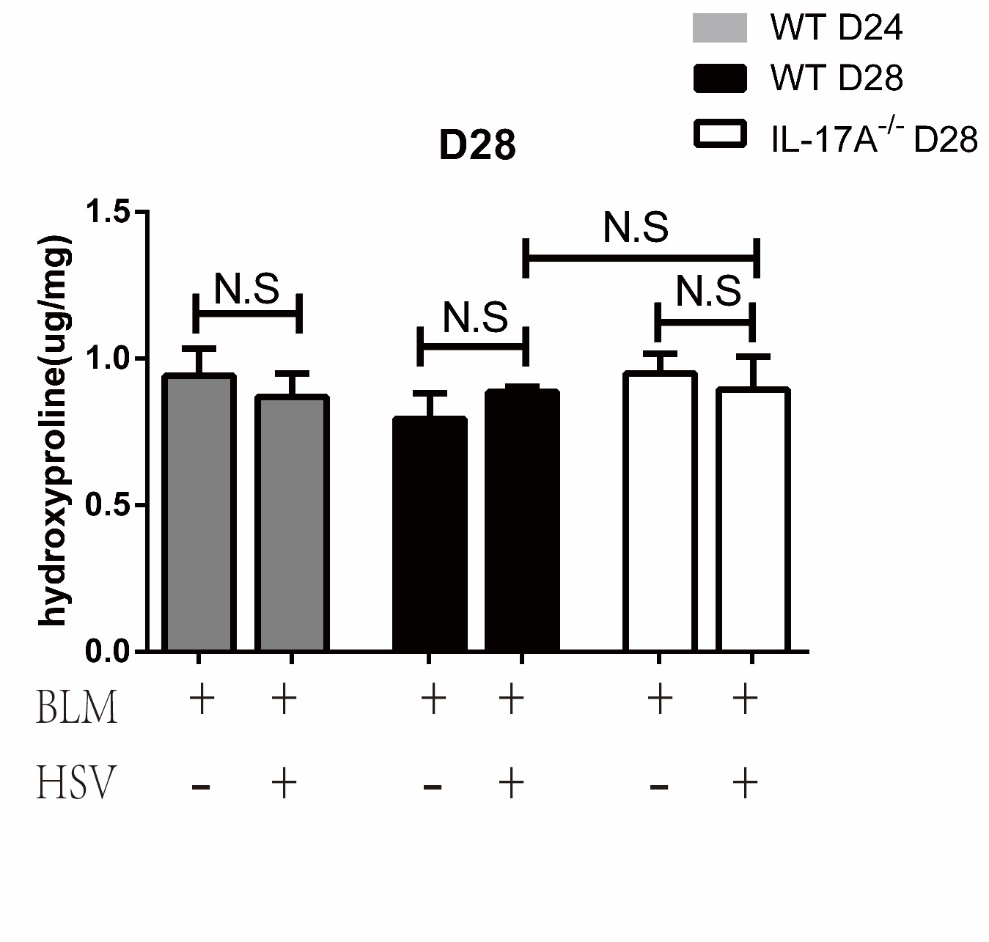
**

**Figure S2 Hydroxy proline measurement of lung tissue.**

WT mice in the BLM+HSV1 and the BLM+Saline groups show similar hydroxyl proline (Day 24: *P*=0.5434; Day 28: *P*=0.3973). No significant difference is in the WT+BLM+HSV1 mice and the IL-17A^-/-^+BLM+HSV1 mice (*P*=0.9341). n=5 in each group. N.S: not significant.


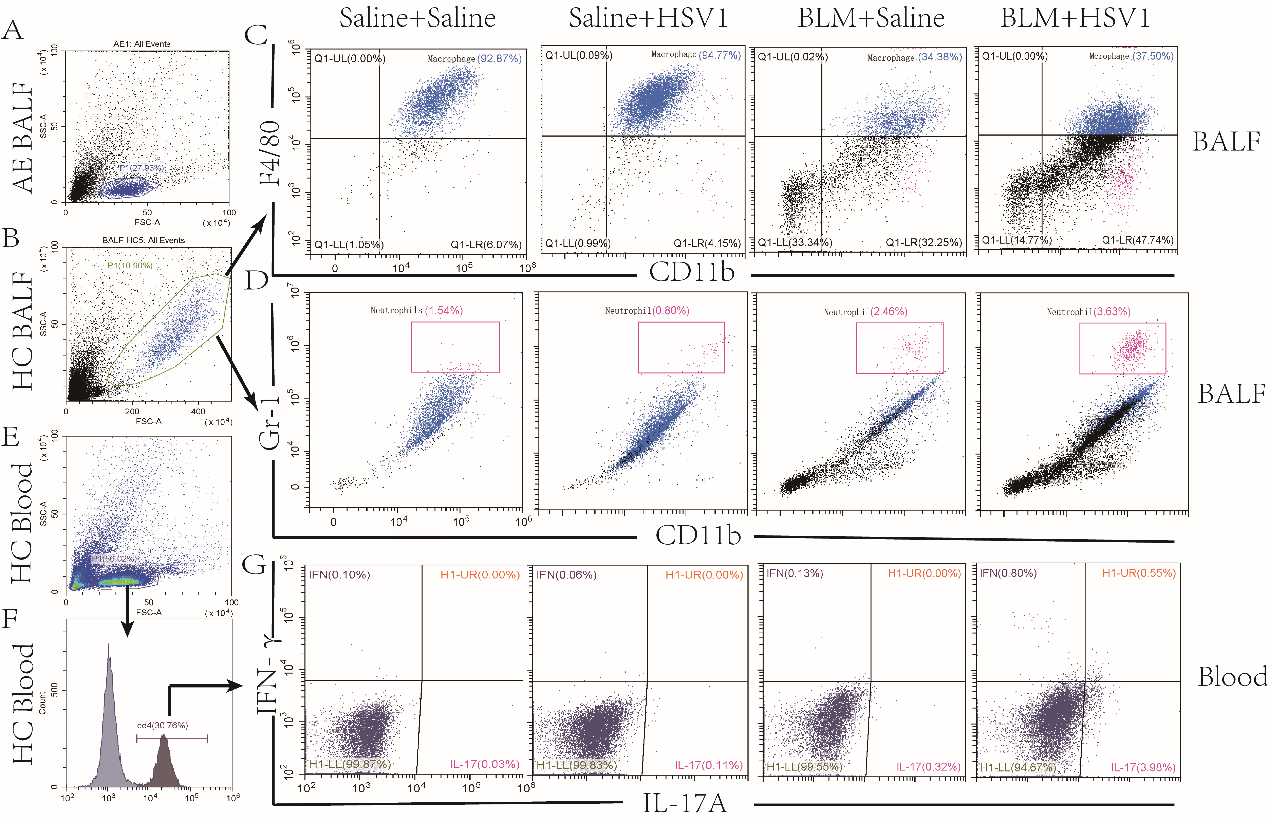


**Figure S3 Flow cytometry of mononuclear cells from BALF and peripheral blood.**

**A and B**. Lymphocytes and granulocyte-macrophages in BALF were gated by their features of FSC-A and SSC-A.

**C and D**. All cells came from Figure B P1. Macrophages and neutrophils and inflammatory monocytes were defined as CD11b^+^+F4/80^+^ and CD11b^+^+Gr-1^+^ respectively.

**E and F.** Mononuclear cells from peripheral blood were stained by CD4-FITC, IFN-γ-APC and IL-17-PE. Lymphocytes were sorted by the features of FSC-A and SSC-A in P1(E). Then CD4+ lymphocytes were isolated by FITC-labeled antibodies(F) from P1 in Figure E.

**G.** All elements were from CD4+ lymphocytes in Figure F. INF-γ and IL-17A were used to label TH1 and TH17 cells, respectively.


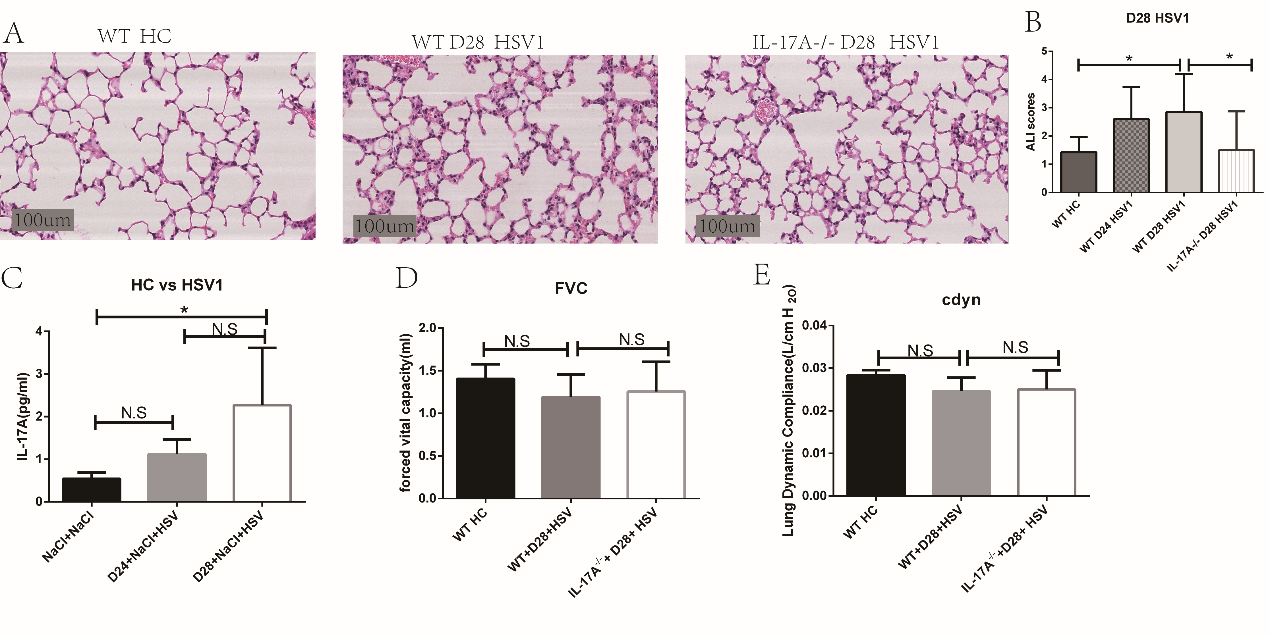


**Figure S4 IL-17A is pathogenic in the HSV1 infection alone mice.**

1. Representative images of H&E staining of the lung tissue of mice in each respective group (200× magnification, scale bar=100 μm). The pathological results demonstrated that WT+HSV1 group had obvious infiltrations of inflammatory cells in the alveolar septum after 7 days of HSV1 infection(D28), and IL-17A^-/-^+HSV1 mice showed less inflammatory cell infiltration in the lung tissue.
2. Mouse lung tissue ALI score. WT+HSV1 group had significantly higher ALI scores than WT+HC group (Figure S4B: WT+D28+HSV1 *vs*. WT+HC, *P*=0.0289). IL-17A^-/-^+HSV1 mice showed lower ALI scores than WT+HSV1mice (WT D28 HSV1 *vs.* IL-17A-/- D28 HSV1, *P*=0.0389).
3. IL-17A concentration in BALF. Compared with WT+HC group, the WT+HSV1 group demonstrated significantly increased IL-17A on post-HSV1 infection day 7 (*P*=0.0195).

**D and E.** Lung function (FVC and Cdyn) of mice HSV1 infected alone. The FVC and Cdyn didn’t show difference after HSV1 infection in WT and KO mice.


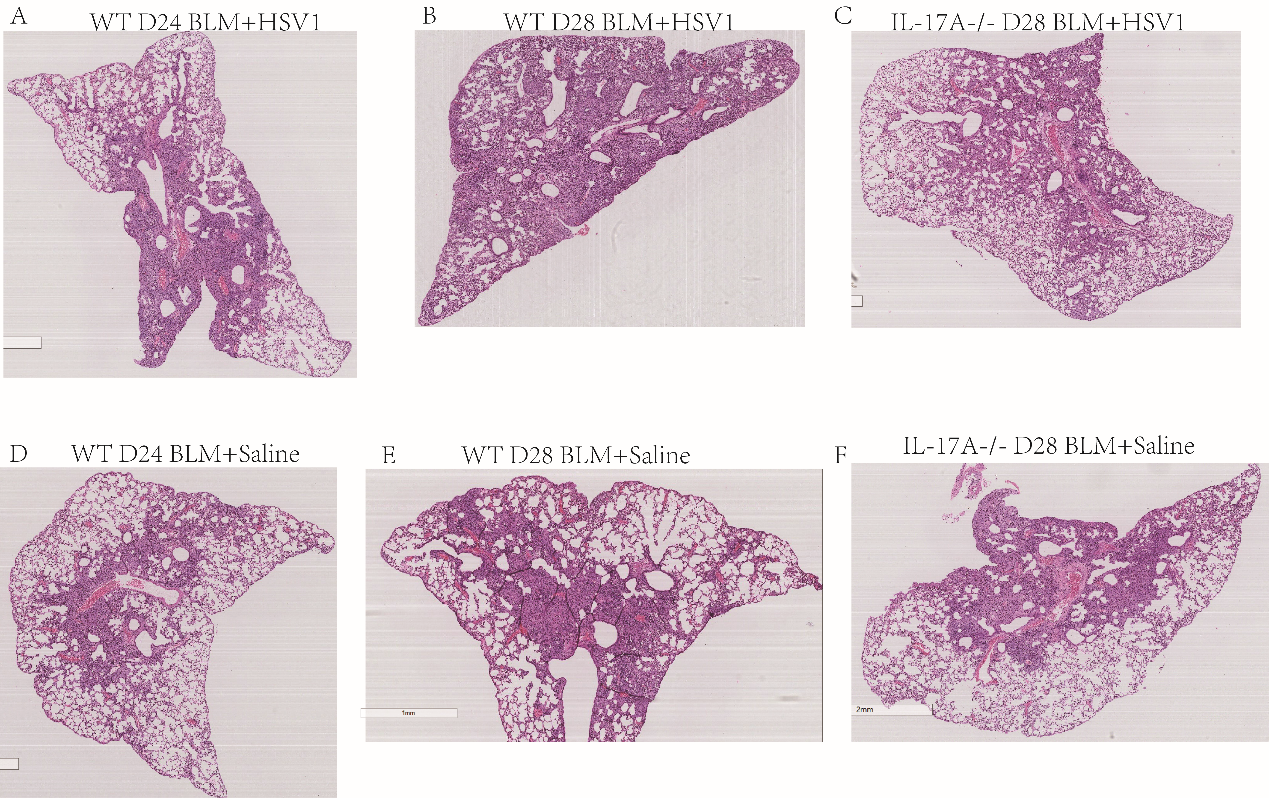


**Figure S5** Lower magnified pathological pictures of H&E staining of the lung tissue of mice in each respective group.


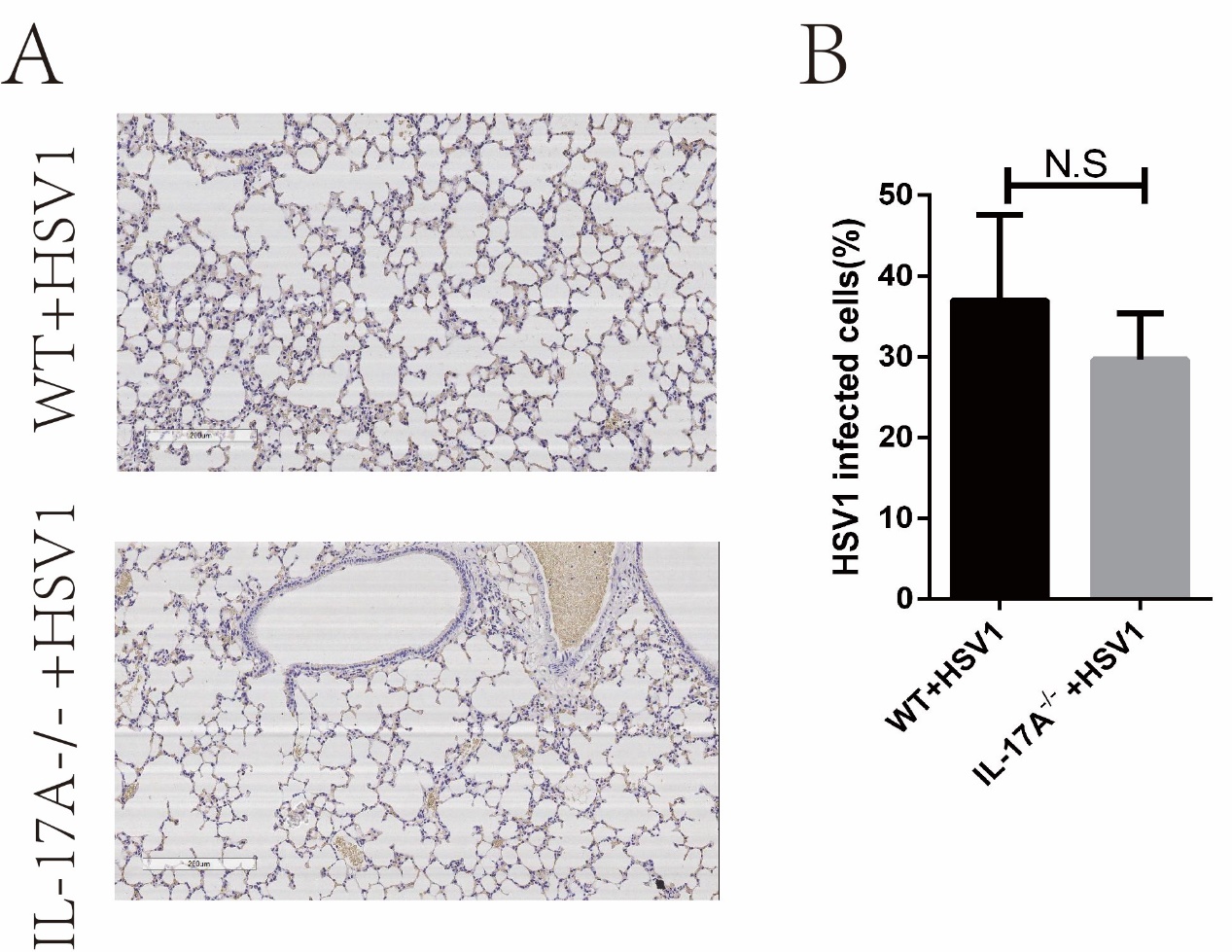


**Figure S6** Immunohistochemical results of lung tissues on post-HSV1 infection day 7.

**A**．Representative images of immunohistochemical results of lung tissues on day 7 post-HSV1 infection (100× magnification, scale bar=200 μm).

**B.** Percentages of cells infected by HSV-1 were quantified.
